# Vascular endothelial growth factor ameliorated palmitate-induced cardiomyocyte injury via JNK pathway

**DOI:** 10.1007/s11626-021-00616-z

**Published:** 2021-11-17

**Authors:** Shi-ya Wang, Cao Zou, Xiao-feng Liu, Yon-jin Yan, Shun-zhon Gu, Xun Li

**Affiliations:** 1 Department of Cardiology, The First Affiliated Hospital of Soochow University, 188 Shizi Street, Suzhou, , Jiangsu China; 2 Hai’an People’s Hospital, Nantong, China

**Keywords:** Palmitate, VEGF, Cardiomyocyte, Apoptosis, Caspase 3, NF-κB

## Abstract

Enhanced apoptosis of cardiomyocytes in suffering overloaded saturated fatty acids (SFAs) can result in myocardial infarction and cardiac dysfunction. The function of vascular endothelial growth factor (VEGF) in cardiomyocyte protection was not clearly described. To investigate the preservative effects of VEGF sensitization on ceramide-mediated programmed cell death of cardiomyocytes, palmitate-induced injury in H9c2 cells was established as an in vitro model. Results revealed that 0.5 mM palmitate application effectively led to debased viability and activated apoptotic factors. A significant time-dependent relation between PAL and cardiomyocyte injury was observed. The apoptosis rate was increased greatly after 16 h of treatment with 0.5 mM PAL. In addition, cell viability was restored by VEGF overexpression during treatment with 0.5 mM PAL. Reduced apoptosis rate and expression of caspase 3, Bax, and NF-κB p65 were observed in this process, while boosted Bcl-2, p-JNK/JNK expression and activity of caspase 3 were checked. However, p-ERK/ERK levels did not exhibit a significant change. These findings indicated the protective effects of VEGF in confronting the ceramide-induced cardiomyocyte apoptosis, and would devote therapeutic targets for cardiovascular safeguard in dealing with fatty acid stress.

## Introduction

Recent studies support the hypothesis that cardiomyocytes harbor no ability of self-renewal and replication, for they are terminally differentiated cells (Heallen *et al*. 2019). Thus, it is believed that the damage and apoptosis of cardiomyocytes are mainly ascribed to cell necrosis (Nasser *et al*. 2020). However, the latest research shows that cardiomyocyte apoptosis is also an important biological basis for the normal physiological function of the heart (Heallen *et al*. 2019). The rapid changes in the human diet, and the concomitant increase in human fat deposition, have led to a high incidence of obesity, diabetes, and cardiovascular diseases (Li *et al*. 2020). Increases in serum lipid levels result in damage to and irritation of the cardiovascular system; particularly, ectopic fat deposition places an additional burden on the heart, which can lead to myocardial injury (Lin *et al*. 2020). Energy supply to the adult heart mainly relies on fatty acids; abnormal fatty acid metabolism is often accompanied with heart disease or systemic disease (Cacicedo *et al*. 2005; Zhou *et al*. 2020). It has been reported that acute myocardial ischemia, diabetes, and obesity patients have an increased risk of abnormal deposition of myocardial fat, leading to heart failure (Puzyrenko *et al*. 1999).

Palmitate (PAL), a saturated fatty acid, has been shown to accumulate in cardiomyocytes, vascular smooth muscle cells, hepatic cells, and islet beta cells (Chai and Liu 2007; Quan *et al*. 2014; Gorgani-Firuzjaee *et al*. 2014; Le *et al*. 2020; Geng *et al*. 2020). PAL accumulation in cardiomyocytes can lead to “lipotoxicity” and thus result in cardiomyocyte injury, apoptosis, and heart dysfunction and failure (Kenny and Abel 2019; Tong *et al*. 2019; Li *et al*. 2020; Xiong *et al*. 2020). Transgenic mouse models with abnormal lipid accumulation in the heart demonstrated that an imbalance of lipid uptake and utilization in cardiomyocytes led to apoptosis and contributed to cardiomyopathy (Cheng *et al*. 2004; Chiu *et al*. 2005). PAL was also found to induce apoptosis in a variety of cells in vitro; however, the exact molecular and cellular mechanisms by which fatty acids cause apoptosis remain unclear.

Vascular endothelial growth factor (VEGF), also known as vascular osmotic factor, is a member of the vascular endothelial growth factor family and exerts multiple physiological effects, including proangiogenesis effects, blood vessel dilation, increasing vascular permeability, and promoting cell proliferation, differentiation, and viability. Extensive studies show its role in the fields of diabetes, rheumatoid arthritis, kidney disease, cardiovascular disease, tumor, and central nervous system diseases (Ferrara 2004; Ho and Kuo 2007; Li *et al*. 2016). VEGF induced migration and proliferation of vascular endothelial cells in the process of angiogenesis and promoted the secretion of protease and fibrinogen activator, which stimulated the cells to escape from the matrix by degrading the vascular basement membrane (Oka *et al*. 2014). Platelet-derived growth factors (PDGFs) are secreted by endothelial cells under the stimulation of VEGF, thus maintaining vascular homeostasis (Bowers *et al*. 2020). VEGF-A, one of the five members in the VEGF family, shows the highest specificity and the strongest physiological effects (Harper and Bates 2008; Rennel *et al*. 2008). VEGF directly interacts with mitogens in endothelial cells and thus promotes the permeability of blood vessels, increases blood oxygen supply, and enhances the proliferation of vascular endothelial cells (Huang *et al*. 2020; Song and Finley 2020). Inhibition of extracellular signal–regulated kinase (ERK)–MAPK signaling by PD98059 significantly increased cardiomyocyte apoptosis, caspase 3 activity, and the myocardial defect area, and the effects on apoptosis could be reversed by the administration of fasudil (a Rho kinase inhibitor) (Czabotar *et al*. 2014). The ERK–MAPK pathway promotes the remodeling of myocardial cell morphology in glucocorticoid-induced cardiomyocyte injury (Yang *et al*. 2015).

In this study, we observed the effects of VEGF overexpression on myocardial apoptosis in a PAL-induced cardiomyocyte apoptosis model and detected the changes of the MAPK pathway, in order to explore the mechanisms and protective factors of myocardial apoptosis injury in the cellular and molecular levels.

## Results

### Effects of different PAL concentrations on cell survival and apoptosis

**Figure 1:**
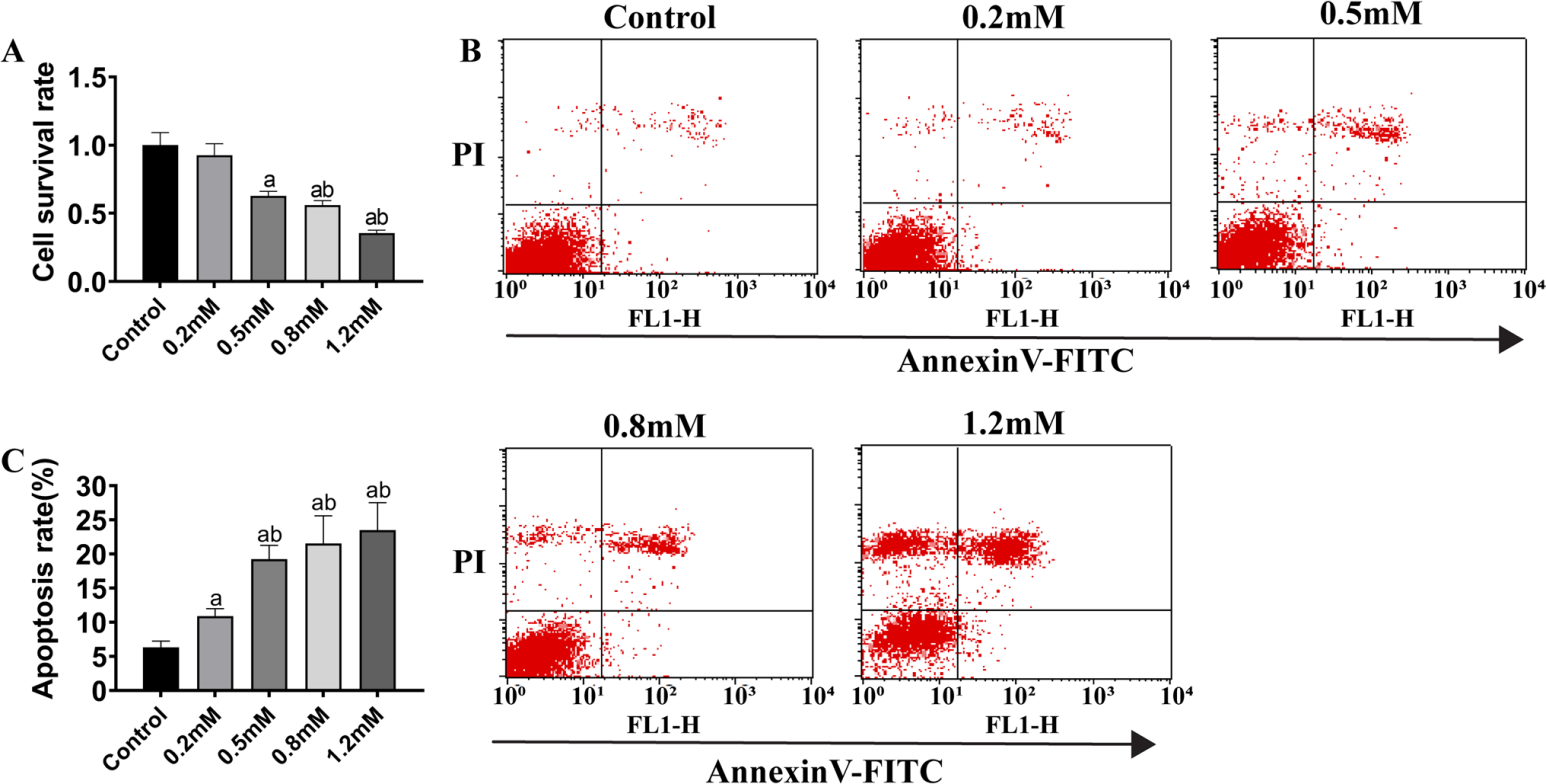
Cell survival and apoptosis after treatment with different PAL concentrations. (***A***) Cell survival rate. (***B***) Flow cytometry analysis of PAL-treated H9c2 cells. (***C***) Statistical analysis of the flow cytometry results. (a) *P* < 0.05 compared with the control group; (b) *P* < 0.05 compared with the 0.2 mM group. *n* = 7 for cell survival investigation and *n* = 3 for FACs analysis

To determine the suitable concentration of palmitate (PAL) in inducing cardiomyocyte cell injury model, H9c2 cells were treated with PAL at final concentrations of 0.2, 0.5, 0.8, and 1.2 mM and collected after 24 h to measure cell viability. Significant decrease was observed in the 0.5 mM group (*P* < 0.05), 0.8 mM group (*P* < 0.01), and 1.2 mM group (*P* < 0.001) when compared with the control group, while significant difference was only detected in the 0.8 mM group (*P* < 0.05) and 1.2 mM group (*P* < 0.01) compared with the 0.2 mM group (Figure 1*A*). To explore the effects of PAL on H9c2 cell apoptosis, the PAL incubated cells above were collected and analyzed by flow cytometry (FACs). The apoptosis rates in the 0.2 mM (*P* < 0.05), 0.5 mM (*P* < 0.01), 0.8 mM (*P* < 0.01), and 1.2 mM (*P* < 0.01) groups were significantly higher than that in the control group. In addition, the apoptosis rates were significantly enhanced in the 0.5 mM (*P* < 0.05), 0.8 mM (*P* < 0.01), and 1.2 mM groups (*P* < 0.01) compared to the 0.2 mM group (Figure 1*B*, *C*). These data indicated that PAL successfully influenced myocardial cell viability and apoptosis in a certain range of concentrations.

### PAL altered expression of apoptosis-related genes

**Figure 2:**
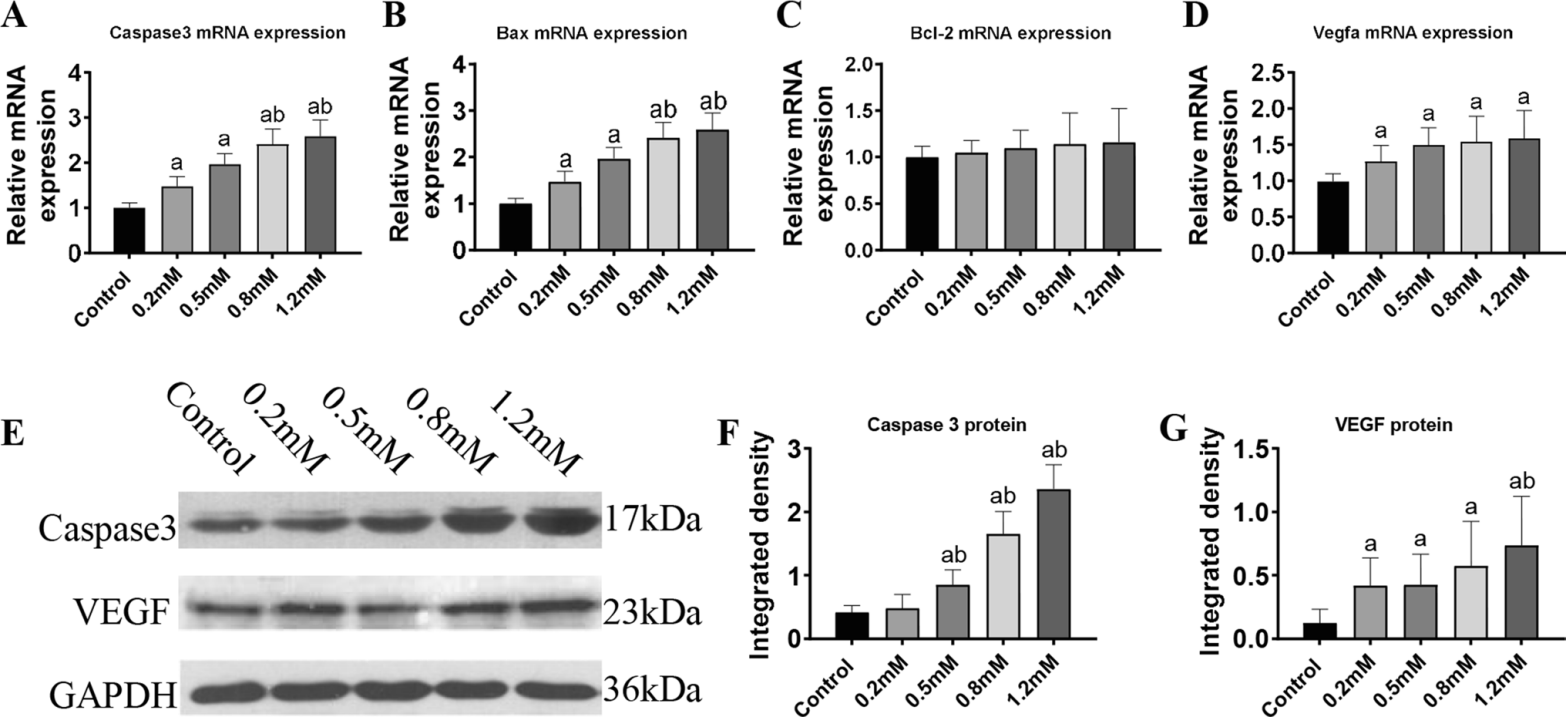
Expression of apoptosis-related genes after treatment with different concentrations of PAL. (***A***)* Casp3* mRNA expression. (***B***) *Bax* mRNA expression. (***C***) *Bcl-2* mRNA expression. (***D***) *Vegfa* mRNA expression. (***E***) Western blot analysis of caspase 3 and VEGF. (***F***) Relative gray density analysis for caspase 3. (***G***) Relative gray density analysis for VEGF. (a) *P* < 0.05 compared with the control group; (b) *P* < 0.05 compared with the 0.2 mM group. *n* = 8 for mRNA detection and *n* = 3 for blots

To investigate the variations of apoptosis-related genes after PAL incubation, H9c2 cells were incubated with PAL at final concentrations of 0.2, 0.5, 0.8, and 1.2 mM for 24 h. Transcriptional alteration of *Casp3* expression enhanced continuously after PAL incubation in the 0.2 mM (*P* < 0.05), 0.5 mM (*P* < 0.01), 0.8 mM (*P* < 0.01), and 1.2 mM (*P* < 0.01) groups when compared with the control group, while its expression was significantly higher in the 0.8 and 1.2 mM groups compared with that in the 0.2 mM group (*P* < 0.05) (Figure 2*A*). In addition, expression of *Bax* was enhanced significantly in the 0.2 mM group (*P* < 0.05) and the 0.5, 0.8, and 1.2 mM groups (*P* < 0.01) compared to that in the control group, while its expression was higher in the 0.8 and 1.2 mM groups compared to that in the 0.2 mM group (*P* < 0.05) (Figure 2*B*). Concurrently, *Vegf*a expression was significantly higher in the 0.2, 0.5, 0.8, and 1.2 mM groups than that in the control group (*P* < 0.05) (Figure 2*D*). However, *Bcl-2* expression was not significantly different in the 0.5, 0.8, and 1.2 mM groups compared with the 0.2 mM group (*P* > 0.05) (Figure 2*C*).

To determine the changes of the protein expression of caspase 3 and VEGF, PAL incubated H9c2 cells were collected and had undergone western blot analysis. Results revealed that enhanced caspase 3 expression was checked in the 0.5, 0.8, and 1.2 mM groups compared to the control group (*P* < 0.05, *P* < 0.01, and *P* < 0.001, respectively). Furthermore, caspase 3 levels were significantly higher in the 0.5, 0.8, and 1.2 mM groups compared to that in the 0.2 mM group (*P* < 0.05, *P* < 0.01, and *P* < 0.001, respectively) (Figure 2*E*, *F*). Similarly, a remarkable increase in VEGF expression was observed in the 0.8 and 1.2 mM groups compared with the control group (*P* < 0.05), while no significant difference was detected compared with the 0.2 mM group (*P* > 0.05). In conclusion, the expression of apoptosis-related genes was enhanced in a dose-dependent way after PAL stimulation for 24 h.

### Effects of different incubation times with palmitate on cell survival and gene expression

**Figure 3:**
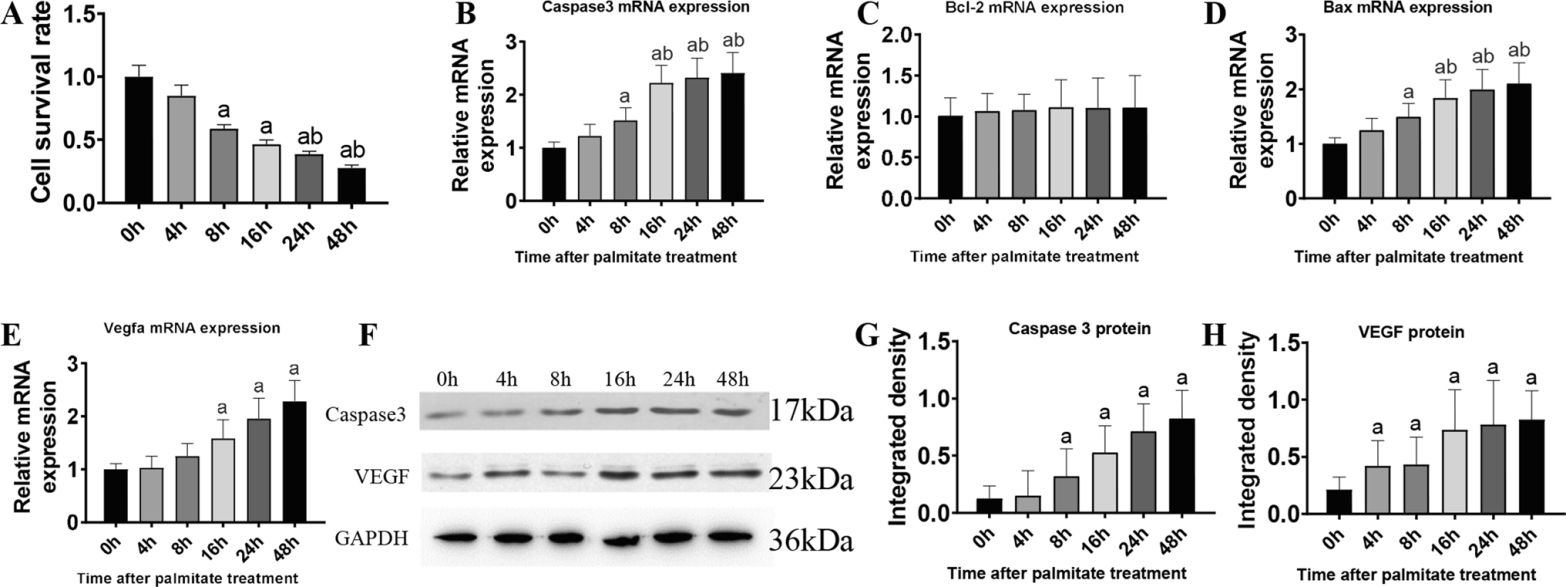
Incubation time of cardiomyocytes with PAL influenced cell survival and the expression of apoptotic genes. (***A***) Cell survival rate. (***B***) *Casp3* mRNA expression. (***C***) *Bcl-2* mRNA expression. (***D***) *Bax* mRNA expression. (***E***) *Vegfa* mRNA expression. (***F***) Western blot analysis of the expression of caspase 3 and VEGF. (***G***) Relative gray density analysis for caspase 3. (***H***) Relative gray density analysis for VEGF. (a) *P* < 0.05 compared with the control group; (b) *P* < 0.05 compared with the 4 h group. *n* = 8 for mRNA detection and *n* = 3 for blots

Considering the optimal effects on gene expression and cell viability, based on the above results, a PAL concentration of 0.5 mM was chosen for subsequent experiments. To analyze the effects of different incubation times on cell injury and apoptosis, cells were divided into the 0, 4, 8, 16, 24, and 48 h groups, and cell viability at each time point was examined by MTT assay. Results showed that a significant decrease was observed at 8 h (*P* < 0.05), 16 h (*P* < 0.01), 24 h (*P* < 0.01), and 48 h (*P* < 0.01) compared to the 0 h group, and it was significantly decreased at 24 h and 48 h compared to the 8 h group (*P* < 0.05) (Figure 3*A*).

To detect the variations of apoptotic-related genes after incubation of PAL at different time points, mRNA and protein of H9c2 cells were analyzed. The transcriptional expression of *Casp3* was significantly higher at 8 h (*P* < 0.05) and 16 h, 24 h, and 48 h (*P* < 0.01) compared to 0 h (Figure 3*B*). The expression of *Bcl-2*, an antiapoptotic gene, showed a slight increase at different time points, but no statistical difference was observed (*P* > 0.05) (Figure 3*C*). The expression of *Bax*, a proapoptotic gene, was significantly higher at 8 h (*P* < 0.05), and16 h, 24 h, and 48 h (*P* < 0.01) compared to the control group (Figure 3*D*). *Vegfa* expression at 16 h (*P* < 0.05), and 24 h and 48 h (*P* < 0.01) was significantly increased compared to the 0 h group (Figure 3*E*).

Western blot analysis was taken to investigate the expression of caspase 3 and VEGF in PAL incubated H9c2 cells. Expression of caspase 3 was significantly enhanced in 8 h, 16 h, 24 h, and 48 h than that in the control group (*P* < 0.05, *P* < 0.01, *P* < 0.01, and *P* < 0.001, respectively), while its expression was significantly higher in the 16, 24, and 48 h groups compared to that in the 4 h group (*P* < 0.01) (Figure 3*F*, *G*). VEGF protein expression also showed a time-dependent increasing trend. A significant increase was observed in the 4 h and 8 h groups compared to the 0 h group (*P* < 0.05). At 16 h, VEGF expression was significantly higher than that in the 4 h and 8 h groups (*P* < 0.01) (Figure 3*F*, *H*).

In summary, the apoptosis rate exhibited a time-dependent increase after PAL stimulation and the expression of apoptosis-related genes and VEGF was enhanced.

### VEGF overexpression ameliorated cell injury

**Figure 4:**
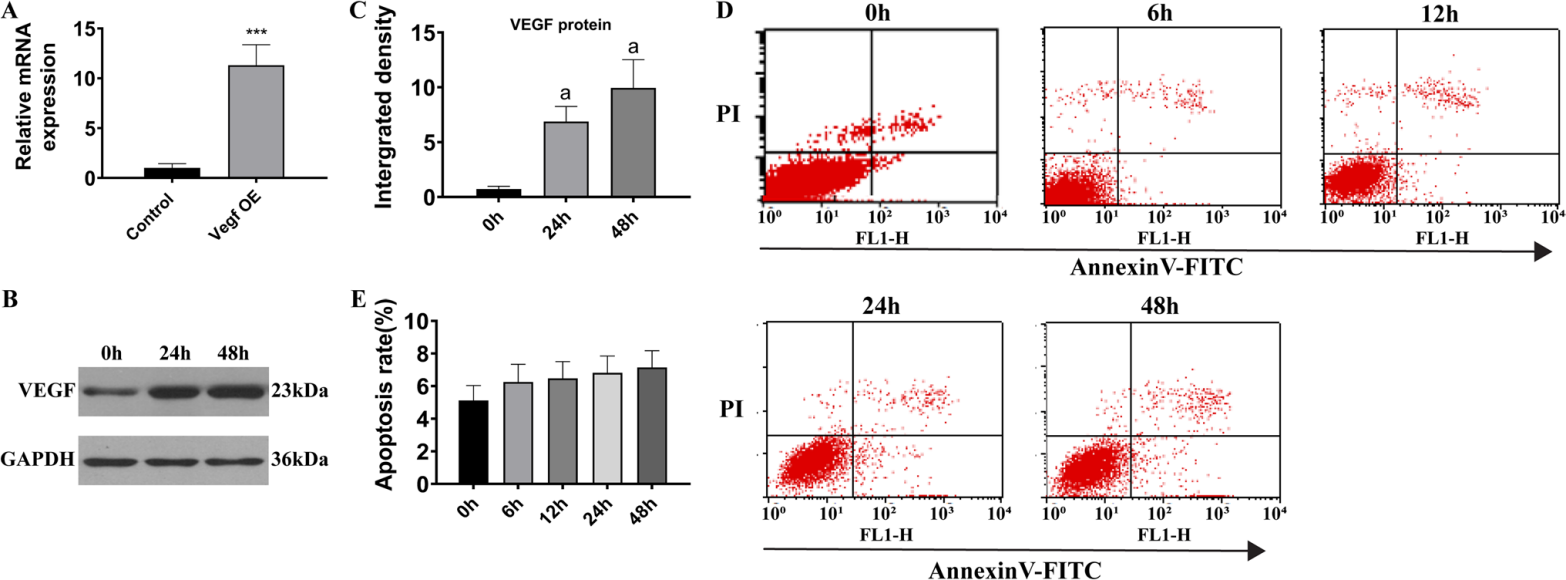
VEGF overexpression reduced the apoptotic effects of PAL. (***A***) VEGF mRNA expression after exotic transfection. (***B***) Western blot analysis of VEGF. (***C***) Relative gray density analysis for VEGF. (***D***) Flow cytometry analysis of the apoptosis of cardiomyocytes. (***E***) Statistical analysis of FACS results. (a) *P* < 0.05 compared with the control group. *n* = 3

To explore the role of VEGF in the regulation of the apoptosis process, a plasmid containing human *VEGFA* cDNA was constructed and administered to H9c2 cells. Results showed that the expression of VEGF-A was boosted at the transcriptional level (48 h; Figure 4*A*) and the translational level (24 h, 48 h; Figure 4*B*, *C*). Moreover, no significant difference in cell apoptosis rate was found by flow cytometry (*P* > 0.05) (Figure 4*D*, *E*).

**Figure 5:**
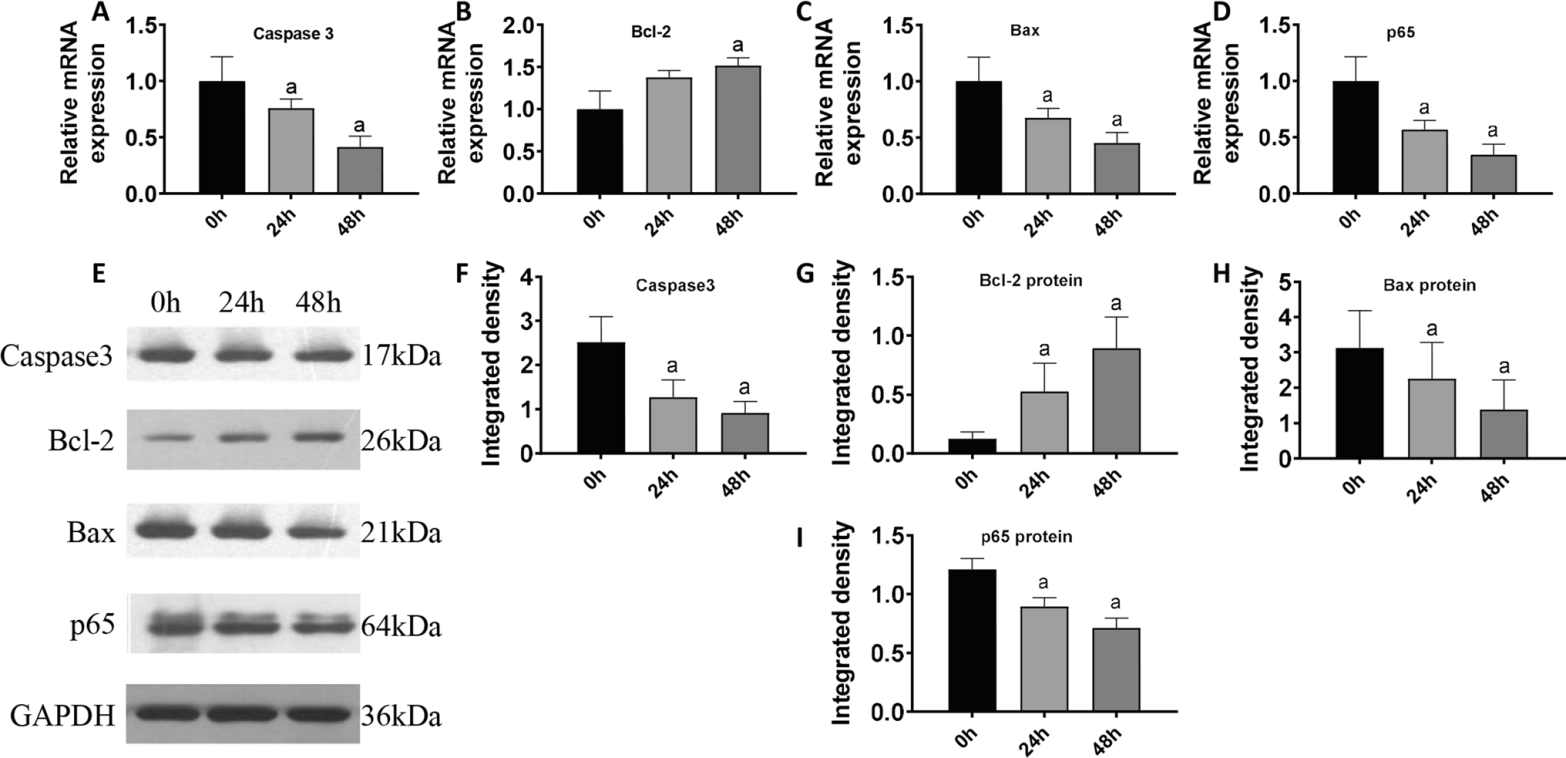
VEGF overexpression reversed the apoptosis of cardiomyocytes. (***A***–***D***) Transcriptional changes of *Casp3*, *Bcl-2*, *Bax*, and *p65* after VEGF overexpression in PAL incubated cardiomyocytes*. *(***E***) Protein expression of caspase 3, Bcl-2, Bax, and p65. (***F***–***I***) Relative gray density analysis for caspase 3, Bcl-2, Bax, and p65 in (***E***). (a) *P* < 0.05 compared with the control group. *n* = 8 for mRNA detection and *n* = 3 for blots

We then measured the expression levels of apoptosis-related factors at 0, 24, and 48 h. The expression level of *CASP3*, a key executor of the apoptotic pathway, was significantly decreased in the 24 h group (*P* < 0.05), and its expression level decreased significantly after 48 h of incubation with PAL (*P* < 0.01) (Figure 5*A*).

A similar decreasing trend was observed for caspase 3 protein expression at 24 h (*P* < 0.05) and 48 h (*P* < 0.05) (Figure 5*E*, *F*). Compared with the control group, the expression level of the antiapoptotic factor *Bcl-2* exhibited no significant difference in the 24 h group (*P* > 0.05), while it was significantly increased at 48 h (*P* < 0.05) (Figure 5*B*). On the contrary, Bcl-2 protein expression was significantly higher than that of the control group at 24 h (*P* < 0.05) and 48 h (*P* < 0.01) (Figure 5*E*, *G*).

Compared with the control group, expression of the proapoptotic factor *Bax* significantly decreased in the 24 h group (*P* < 0.05) and the 48 h group (*P* < 0.01) (Figure 5*C*). Bax protein expression declined at 24 h (*P* < 0.05) and 48 h (*P* < 0.01) (Figure 5*E*, *H*). Expression of *p65*, encoding a subunit of the NF-κB transcriptional complex, was significantly decreased at 24 h (*P* < 0.05) and 48 h (*P* < 0.01) compared with the control group (Figure 5*D*). Meanwhile, p65 protein expression was significantly lower at 24 h (*P* < 0.05) and 48 h (*P* < 0.01) (Figure 5*E*, *I*).

In summary, VEGF-A overexpression increased the cell survival rate and reduced the apoptosis rate after PAL incubation, and VEGF-A overexpression significantly lowered the expression of apoptosis-related factors.

### The JNK pathway, but not the ERK pathway, functioned in VEGF-regulated cell apoptosis

**Figure 6:**
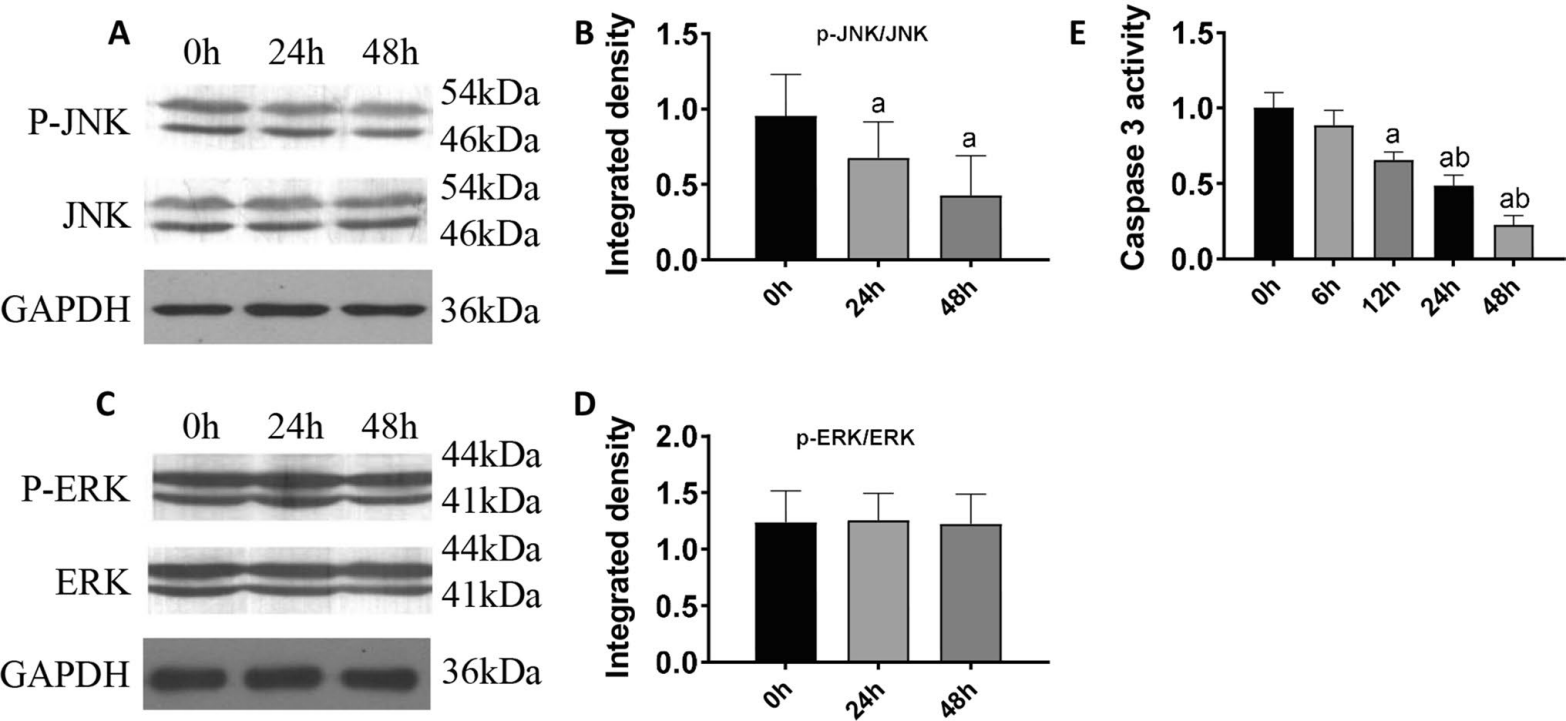
Variation of the JNK and ERK pathways under condition of VEGF overexpression. (***A***) Protein levels of p-JNK and JNK. (***B***) Relative gray density analysis for p-JNK and JNK. (***C***) Protein levels of p-ERK and ERK. (***D***) Relative gray density analysis for p-ERK and ERK. (***E***) Enzyme activity of caspase 3. (a) *P* < 0.05 compared with the control group; (b) *P* < 0.05 compared with the 0.2 mM group. *n* = 3 for blots and *n* = 55 for enzyme activity analysis

Studies have shown that the c-Jun N-terminal protein kinase (JNK) and ERK pathways play important regulatory roles in the process of apoptosis. Therefore, we detected their protein expression. The expression level of phosphorylated JNK (p-JNK) was significantly lower in the 24 h and 48 h groups than in the control group (*P* < 0.05) (Figure 6*A*, *B*); no significant difference was observed in the JNK protein level at 24 and 48 h (*P* > 0.05) (Figure 6*A*, B). However, the expression level of phosphorylated ERK (p-ERK) was not significantly different in the 24 and 48 h groups (*P* > 0.05) (Figure 6*C*, *D*), and there was no significant difference in total ERK protein expression between the 24 h and 48 h groups (*P* > 0.05) (Figure 6*C*, *D*).

After H9c2 cells were transfected with VEGF-A plasmid and control cells were transfected with empty vector, cells were incubated with 0.5 mM PAL for different periods of time and caspase 3 activity was evaluated with the caspase 3 activity assay kit. The results showed that caspase 3 activity in the 12 h group was significantly lower than that in the control group (*P* < 0.05), and caspase 3 activity in the 24 and 48 h groups was significantly lower than that in the control group (*P* < 0.01) (Figure 6*E*).

Totally, JNK activation, instead of ERK, was involved in the function of VEGF in antagonizing the proapoptotic effects of PAL and ectopic expression of VEGF countered the activity of caspase 3.

## Discussion

Cardiovascular disease has become the number one challenge endangering human health in today’s society according to the World Health Organization (Rodriguez *et al*. 2014). The age-adjusted prevalence of total heart diseases is 10.6% based on the 2017 National Health Interview Survey, and the prevalence is 11.0%, 9.7%, 7.4%, and 6.1% among whites, blacks, Hispanics, and Asians, respectively (Virani *et al*. 2020). Statistics from the USA show that in 2018, about 163.6 people per 100,000 standard population died of heart disease, making heart disease the leading cause of death (Xu *et al*. 2018a,  2020). In the present study, an in vitro model of lipotoxicity-induced cell injury was successfully constructed. After incubation with 0.5 mM PAL for 24 h, cell viability was significantly reduced, apoptosis was upregulated, and VEGF expression was increased. After transient transfection with VEGF, VEGF overexpression ameliorated lipotoxicity-induced cell injury and lowered the activity of JNK.

Ectopically deposited fatty acids in the heart result in lipotoxicity, injury to cardiomyocytes, and myocardial apoptosis (Li *et al*. 2020). PAL, also known as hexadecane, is a commonly consumed saturated fatty acid. Numerous studies have reported that PAL induced apoptosis in a variety of cells, including Chinese hamster ovary (CHO) cells, rat islet cells, retinal microvascular endothelial cells, and myocardial cells (Sparagna *et al*. 2000; Listenberger *et al*. 2001; Yamagishi *et al*. 2002; Lytrivi *et al*. 2020). A review of the published literature shows that mitochondrial pathways, death receptor pathways, and endoplasmic reticulum pathways are involved in the PAL-induced apoptotic process (Westermann 2010; Vakifahmetoglu-Norberg *et al*. 2017). Overstimulation by PAL causes irreversible alteration of mitochondrial permeability and osmotic pressure, thus inducing caspase 9 to activate caspase 3 and caspase 7 to cleave downstream substrates (Pradelli *et al*. 2010). In addition, Bcl-2 family members play an important role in regulating mitochondrial membrane permeability. Bcl-xl and Bcl-2 are distributed in the mitochondrial membrane and the cytoplasm, whereas Bax and Bid are located in the cytoplasm (Czabotar *et al*. 2014). Mitochondrial damage leads to loss of normal morphology and energy metabolism (Richter *et al*. 1996). Many questions on the relationship between VEGF and PAL in cardiomyocyte apoptosis still remain to be answered. In the present study, the effects of different concentrations and incubation times of PAL on apoptosis in H9c2 cells were determined by cell viability assays and analysis of the expression of apoptotic factors. Cells were cultured with medium containing 0.2, 0.5, 0.8, and 1.2 mM PAL for 24 h to determine the optimal PAL concentration to induce the cell injury model. Furthermore, different incubation times (0, 4, 8, 16, 24, and 48 h) were adopted to screen the appropriate time for the cell injury model construction. In addition, we also detected the levels of the antiapoptotic Bcl-2 and the proapoptotic Bax, as well as the downstream protein caspase 3. Results showed that PAL increased Bax and caspase 3 levels, while it decreased the expression of VEGF, thus promoting cell apoptosis.

VEGF was reported as a vital factor in the promotion of angiogenesis by inhibiting endothelial cell apoptosis upon PAL treatment (Yang *et al*. 2015; Xu *et al*. 2018b). In the present study, we focused on cell survival and apoptosis during PAL incubation to investigate the role of VEGF in cardiomyocytes. Since incubation with PAL boosted the expression of VEGF, we hypothesized that VEGF might play a pivotal role in lipotoxicity-induced myocardial apoptosis. 12-Deoxyphorbol 13-palmitate was reported to inhibit the expression of VEGF in MCF-7 cells, which was inconsistent with the results in the present study, possibly because different cells were used in different studies. Next, VEGF was overexpressed and the effects of VEGF on normal and PAL-treated H9c2 cells were evaluated. The PAL-induced decrease in cell viability was ameliorated by VEGF overexpression by inhibiting Bax and caspase 3 expression, while enhancing Bcl-2 expression.

The JNK/ERK signaling pathway has been reported to be involved in apoptosis. Previous reports indicated that a high fat diet induced activation of JNK, which was reversed by *TLR4* knockout (Hu and Zhang 2017). Cellular accumulation of ceramide activated JNK signaling and apoptosis, which was prevented by ceramide synthase 5 (*CERS5*) knockdown (Leonardini *et al*. 2017). JNK activation was observed in PAL-treated cardiomyocytes and attenuated by protein kinase R (PKR) inhibition (Mangali *et al*. 2019). In the present study, we analyzed the expression of proteins involved in the JNK and ERK pathways in cells overexpressing VEGF after PAL treatment. JNK activity was enhanced after PAL incubation, which was alleviated after VEGF overexpression. However, no significant variation in ERK levels was detected. The results suggest a novel role of VEGF in antagonizing cytotoxicity in cardiomyocytes, indicating a potential therapeutic strategy for cardiac protection.

## Materials and methods

### Cell culture and treatments

The rat embryonic heart–originated H9c2 cell line was purchased from the Institution of Biochemistry and Cell Biology, Chinese Academy of Sciences (Shanghai, China), and maintained with high glucose DMEM supplied with 10% FBS (Gibco, Rockville, MD) in 5% CO_2_ containing air at a 37℃ incubator (Thermo Fisher Scientific, Waltham, MA). Cells were dislocated from the dishes by trypsin and suspended into single cell solution for subsequent tests at 80% confluency.

Cells were seeded in a 24-well plate and treated with different concentrations of palmitate (PAL, Sigma-Aldrich, St. Louis, MO) at final concentrations of 0.2, 0.5, 0.8, and 1.2 mM when the confluence reached 50%. In the time-course experiments, cells were treated with PAL at a final concentration of 0.5 mM for 0, 4, 8, 16, 24, and 48 h. The pcDNA3.1-VEGFA plasmid was gifted by Dr. Yinchuan Li’s lab at Nantong University. Cells were transfected with VEGFA plasmid with Lipofectamine 2000 (Thermo Fisher Scientific) according to the manufacturer’s instructions.

### MTT assay

Cell viability was detected by MTT assay. In brief, cells were seeded into 96-well plates at about 1 × 10^4^ cells/well. Cells were treated with designed concentration of PAL or scheduled time for 0.5 mM PAL incubation after 24 h of culture. At the end of each time point, 0.1 mg MTT (Sigma-Aldrich) was added to each well, and plates were incubated for 4 h at 37 °C away from light. Then, the medium was aspirated and 150 μL dimethyl sulfoxide (DMSO; Sigma-Aldrich) was dropped into each well. After incubation on a rotator for 15 min, the absorbance of the solution of each well was detected at a wavelength of 490 nm using a microplate reader (Thermo Fisher Scientific; Varioskan Flash). The cell viability rate was calculated using the following formula:

Viability rate (%) = 100 × (OD value of experimental group / OD value of control group).

### H9c2 cell apoptosis assessment

Exponentially growing cells were plated in 6-well plates at a density of 1 × 10^5^ cells/well. After culture and treatment, cells were trypsinized and resuspended with binding buffer, and the cell density was adjusted to 1 × 10^5^ cells/mL. Each tube containing 100 μL binding buffer was incubated with 5 μL Annexin V-FITC and 5 μL propidium iodide (PI) for 15 min on ice away from light. An additional 400 μL of binding buffer was added and the samples were analyzed on a flow cytometer (FACS, Beckman Coulter, Pasadena, CA). Each sample was measured in triplicate.

### *qRT-PCR*

Total RNA was extracted from H9c2 cells with TRIzol reagent (Invitrogen, Thermo Fisher, Carlsbad, CA) following the manufacturer’s instructions. The RNA was quantified with a spectrophotometer (NanoDrop 2000, Thermo Fisher Scientific) and then reverse-transcribed with PrimeScript™ RT Master Mix (Takara, Shiga Prefecture, Japan, PR036A). Quantitative real-time PCR (qRT-PCR) was performed with SYBR Premix Ex Taq (Tli RNaseH Plus) (Takara, DRR420A). Measurements were carried out in triplicates and data were normalized to endogenous GAPDH expression. Primers were designed by Primer Express software (Applied Biosystems, Foster, CA) and validated. The sequences of primers used are listed in Table 1. The target genes were amplified by PCR program with 2 stages: stage I: 95℃, 30 s; stage II: 95℃, 5 s; 60℃, 34 s, repeat stage II for 40 cycles.

**Table 1:** Sequences of qRT-PCR primers

Gene name	Forward primer	Reverse primer
*GAPDH*	GCAATGTTGCCAGTGTCTGT	GCCTTGACCTTTTCAGCAAG
*CASP3*	CAGAGGGGATCGTTGTGAAG	CATACAAAGAAGTCGGCCTCCA
*Bax*	GGGATGGCCTCCTTTCCTAC	TTCCAGATGGTGAGTGAGGCA
*Bcl-2*	TTCTTTGAGTTCGGTGGGGTC	TGCATATTTGTTTGGGGCAGG
*VEGF*	ACTTTCTGCTGTCTTGGATG	CTCGGCTTGTCACATCACCG
*p65*	CCCCACGAGCTTGTAGGAAAG	CCAGGTTCTGGAAACTGTGGAT

The expression of each gene was defined as the fold change compared with the threshold cycle (Ct), and relative expression levels were calculated using the 2^−∆∆Ct^ method by normalization to the housekeeping gene *GAPDH*. Results were presented as the mean from three independent experiments.

### Western blot

Cells were harvested at certain time points with pre-cooled phosphate-buffered saline (PBS) and washed three times. Cells were in situ incubated with RIPA lysis buffer on ice for 30 min. The lysates were pipetted and centrifuged at 12,000* g* at 4 °C for 15 min. The supernatant was collected and protein concentrations were determined using a BCA kit. Total protein (20–100 μg per lane) was separated by SDS-PAGE and transferred to a polyvinylidene difluoride (PVDF) membrane (Millipore, Billerica, MA). The membrane was blocked in 5% skim milk solution and incubated with anti-VEGF (ab1316), anti-caspase 3 (ab13847), anti-Bcl-2 (ab692), anti-Bax (ab32503), anti-p65 (ab16502), anti-p-JNK (ab124956), anti-JNK (ab208035), anti-ERK (ab184699), anti-p-ERK (ab201015), or anti-GAPDH (ab181602) at 4 °C overnight. Then, membranes were washed and incubated with HRP conjugated secondary antibodies. Protein bands were visualized with ECL Super Signal (Pierce, Rockford, IL). Images were taken using a Tanon 5200 system (Shanghai, China) and the relative gray density was analyzed by ImageJ software (NIH, NY, USA), using GAPDH as an internal control.

### Caspase 3 activity assay

A sensitive assay kit for the quantification of caspase 3 activity in mammalian cells (K533, BioVision, Milpitas, CA) was taken to the current assay. Briefly, cells were washed and digested by pre-cooled PBS buffer for 10 min. Then, the lysate was incubated at 37 °C for 2 h with 50 μL of 2 × reaction buffer (containing 10 mM dithiothreitol) and 5 μL of DEVD-7-amino-4-trifluoromethylcoumarin (Ac-DEVD-AFC) in each group. The fluorescence intensity of the samples was detected with 400 nm excitation wavelength and 505 nm emission wavelength. Data were collected in triplicate and processed with Microsoft Excel 2016 (Microsoft, San Francisco).

### Statistical analysis

Statistical analysis was performed using SPSS Statistics 24.0 (SPSS Inc., Chicago, IL). All data are presented as mean ± standard deviation ($\overline{x}\pm \mathrm{S }$), and the time-course data such as those from the MTT assay were analyzed by the unpaired Student *t*-test. Differences between groups were analyzed using one-way ANOVA (analysis of variance), followed by Tukey’s test, with significance measured at *P < 0.05, **P < 0.01, or ***P < 0.01.
